# MHENet: a multimodal hybrid enhancement network for accurate classification of unsound maize kernels

**DOI:** 10.3389/fpls.2026.1867535

**Published:** 2026-06-26

**Authors:** Chenxia Wan, Ruitao Li, Wenzheng Li, Peng Li

**Affiliations:** 1 Key Laboratory of Grain Information Processing and Control (Henan University of Technology), Ministry of Education, Zhengzhou, China; 2 Henan Key Laboratory of Grain Storage Information Intelligent Perception and Decision Making, Henan University of Technology, Zhengzhou, China; 3 Institute for Complexity Science, Henan University of Technology, Zhengzhou, China

**Keywords:** multimodal hybrid enhancement network, unsound maize kernels, hyperspectral imaging, multimodal fusion, cross modal feature alignment

## Abstract

Accurate classification of unsound maize kernels is critical for grain quality detection and grading. Single red-green-blue (RGB) images are incapable of effectively capturing variations in the internal chemical components of maize kernels, which easily causes category confusion. Meanwhile, hyperspectral images (HSI) lacks the spatial structural information and exhibits the limited discriminative ability when used independently. To address these challenges, this paper proposes a multimodal hybrid enhancement network (MHENet) with a parallel spectral-spatial dual-branch architecture. The proposed network adopts an optimized 1DCNN-3 module for hyperspectral sequence feature extraction and MobileNetV4-Small for lightweight RGB image spatial feature extraction, enabling the comprehensive capture of both spectral physicochemical characteristics and external morphological appearance features of maize kernels. Specifically, the cross-modal projection embedding (CMPE) module achieves heterogeneous feature space alignment; the bidirectional Mamba attention (BMA) module extracts global contextual information with linear complexity; and the entropy-driven adaptive gating (EAG) fusion module enables dynamic weighted fusion of multimodal features. Experiments are conducted on a self-constructed RGB and HSI dataset of unsound maize kernels. The experimental results demonstrate that the proposed MHENet achieves an accuracy of 97.68%, a macro-P of 97.61%, a macro-R of 97.28%, and a macro-F1 of 97.44% in six-category maize kernel classification. Five-fold cross-validation results in an average accuracy of 97.62% and a standard deviation of 0.14. Compared with feature concatenation, Hadamard product, and cross-attention fusion strategies, the proposed MHENet outperforms these methods in terms of accuracy, lightweight, and stability. This work provides an essential guidance to accurately classify unsound maize kernels.

## 1 Introduction

Unsound grains serve as a core indicator for evaluating grain quality levels. During the entire process of growth, harvesting, storage, and processing circulation, grains are prone to defects induced by pests, diseases, mechanical damage, thermal damage, mold growth, and other factors, which directly impair the safety of grain storage, processing efficiency, and commodity value (Zhang et al., 2024; Li et al., 2026; Zhang et al., 2022). As one of the most important grain crops in China, maize plays a crucial role in national food security, thus, the rapid and accurate classification of unsound maize kernels has important engineering application value for grain purchasing, storage, grading, intelligent sorting, and quality control (Shao et al., 2020; Zhu et al., 2024). Traditional manual detection methods rely on visual inspection, which suffered from low efficiency, high labor intensity, strong subjectivity, and poor consistency, making it challenging to meet the large-scale, automated, and high-precision detection requirements of the modern grain industry. In this context, intelligent detection technologies based on machine vision and deep learning have gradually become the mainstream solution, providing a feasible technical approach for the efficient recognition of unsound maize kernels (Wang et al., 2025; Chang et al., 2026).

In recent years, deep learning-based recognition methods using RGB images have been widely applied to grain defect detection tasks (Zhao et al., 2025). Benefiting from powerful local feature modeling capabilities, convolutional neural networks (CNNs) can effectively extract grain visual characteristics, including external morphology, surface texture, and color distribution, achieving promising performance in conventional grain defect recognition (Qiao et al., 2026). However, these methods solely rely on surface visual information and are insensitive to deep-seated defects (e.g., changes in internal grain components, tissue degeneration, and early mold infestation), often leading to misclassification of grains with similar appearances but significant internal differences (e.g., disease-spotted, mold-damaged, and heat-damaged grains), thus failing to meet the requirements of practical quality inspection (Fan et al., 2026). HSI technology can capture continuous spectral reflectance information that sensitively reflects the internal chemical composition, starch structure, and disease severity of grains (Meng et al., 2025), thereby providing an effective discriminative basis for visually similar samples. Nevertheless, HSI inherently lacks spatial geometric structure and texture distribution information, making it insufficient to comprehensively characterize the overall attributes of grains when utilized independently. Additionally, hyperspectral data suffers from high data redundancy and inherent difficulties in modeling long-range spectral dependencies, which severely restrict the improvement of model recognition robustness in practical detection tasks (Sun et al., 2025). Therefore, effectively integrating the spatial structural advantages of RGB images with the physicochemical characterization capabilities of HSI, and developing a multimodal recognition model that balances fine-grained discriminative performance and lightweight inference efficiency, has become an urgent and critical research challenge for advancing the intelligent detection of unsound maize kernels.

To address the aforementioned challenges, this paper constructs a bimodal maize kernel dataset comprising of RGB visible light and HSI data, and develops a multimodal hybrid enhancement network (MHENet) to realize collaborative enhancement and adaptive fusion of multimodal features. The main contributions of this paper are summarized as follows:

1. We propose a MHENet, which adopts a spectral-spatial dual-branch parallel architecture. Specifically, an optimized 1DCNN-3 is designed for hyperspectral sequence feature extraction, and MobileNetV4-Small is employed for lightweight RGB image feature extraction. While fully capturing the spectral physicochemical features and spatial appearance features of maize kernels, the network significantly reduces model parameters and computational overhead, achieving simultaneous improvements in the accuracy of unsound maize kernel recognition and model lightweight.
2. We construct a cross-modal projection embedding (CMPE) module to achieve heterogeneous feature space alignment, propose a bidirectional Mamba attention (BMA) module to extract global contextual information with linear complexity, and design an entropy-driven adaptive gating (EAG) fusion module to realize dynamic weighted fusion of multimodal information. The synergistic cooperation of the three modules significantly enhances the model’s discriminative ability for fine-grained and easily confused maize kernel defects, such as subtle lesions and early mold contamination.
3. We conduct experimental verification on the constructed RGB and HSI bimodal dataset of unsound maize kernel. The results demonstrate that the proposed MHENet significantly outperforms existing mainstream models in terms of recognition accuracy and inference speed. While maintaining a lightweight design, the proposed method achieves higher recognition accuracy and faster inference speed, thereby verifying the effectiveness and superiority of the proposed method.

The structure of this paper is organized as follows. Section 2 reviews relevant work, including machine learning and deep learning methods for classifying unsound maize kernels. Section 3 details the overall framework and core components of the proposed MHENet. Section 4 introduces the experimental datasets, parameter settings, followed by a detailed discussion of the experimental results and performance analysis. Section 5 summarizes the important conclusions.

## 2 Related work

### 2.1 Machine learning based methods for classifying unsound maize kernels

Research on crop seed detection and classification can be traced back to the early research stage. Prior to the widespread application of deep learning, related studies primarily relied on image processing technologies and machine learning methods to accomplish seed recognition and grain quality analysis (Ran et al., 2026). Zhu et al. (2024) developed a recognition method based on online near-infrared spectroscopy and machine learning to distinguish between new and aged maize seeds. Shen et al. (2026) proposed the WC-SVM method by integrating modified watershed segmentation, convex hull defect detection, and a SVM classifier, achieving effective segmentation and damage recognition of maize kernels. Fan et al. (2024) proposed a damage rate prediction model based on machine vision and machine learning algorithms. To this end, a new phenotypic feature dataset of high-moisture maize kernels was constructed by extracting seven phenotypic features. Subsequently, mainstream machine learning algorithms were employed to establish regression models for predicting kernel weight and classification models for kernel defect detection. This method enabled rapid defect classification and accurate prediction of broken kernel weight, thereby achieving the goal of quantitative detection of the breakage rate. Yu et al. (2025) proposed a maize kernel quality prediction model based on machine vision and machine learning algorithms to identify broken kernels. A phenotypic feature dataset of maize kernels was constructed by extracting geometric features. Afterwards, a regression model for maize kernel quality was established using popular machine learning algorithms, achieving quantitative prediction of maize kernel quality.

Previous relevant studies have successfully combined near-infrared spectroscopy and machine vision technology to construct diverse detection models, which have been widely applied in distinguishing new and aged maize seeds, identifying grain damage degrees, predicting kernel damage rates, and realizing the quantitative evaluation of overall maize kernel quality. Nevertheless, such methods heavily rely on manual feature engineering and complicated data preprocessing procedures. In practice, the defect characteristics of unsound maize kernels are extremely complex and diverse. Various defective categories only present subtle differences in local texture, tiny surface cracks, and slight color variation. As a result, manually designed shallow handcrafted features fail to effectively capture such fine-grained discriminative information, limiting the overall detection accuracy and generalization performance of traditional machine learning-based methods.

### 2.2 Deep learning based methods for classifying unsound maize kernels

With the rapid advancement of deep learning theory and computing platforms, deep learning-based methods have gradually become the main research direction for grain quality inspection (Luo et al., 2026; Xu et al., 2026). In particular, the successful application of CNNs in tasks such as image classification, object detection, and semantic segmentation has provided a new technical approach for grain quality detection (Deng et al., 2026). Bi et al. (2022) improved maize seed recognition accuracy by combining deep learning with machine vision and leveraging the Swin Transformer as the backbone. Yang et al. (2024) designed a maize kernel detection and recognition model named YOLOv7-MEF, using YOLOv7-Tiny as the backbone network. Zhang et al. (2025) proposed the LightMCS lightweight recognition model to address the challenge of balancing model accuracy and computational cost in maize kernel quality recognition. Zhao et al. (2025) proposed a lightweight DenXt network to solve issues such as large parameter counts, slow inference speeds, and limited deployability in deep learning models. Wang et al. (2025) proposed a maize kernel detection technology that integrates deep learning and sliding window technology.

Nevertheless, all the aforementioned methods rely solely on single RGB image data, which can only capture external surface features and fail to perceive subtle variations in the internal chemical components of grains. This inevitably causes severe category confusion among fine-grained defective maize kernel samples. To mitigate this limitation, researchers have introduced hyperspectral imaging technology into the classification task of unsound maize kernels to complement intrinsic spectral characterization information. Lv et al. (2022) explored a detection method that integrated HSI with a self-architectural search deep network to classify wheat kernels with different degrees of Fusarium head blight damage. Ageh et al. (2025) comprehensively analyzed six mainstream optical imaging technologies for non-destructive detection of grain quality, and the results indicated that the integrated application of multi-optical imaging technology can effectively optimize grain quality monitoring and postharvest management. Kim et al. (2026) developed an AI-driven diagnostic framework using a custom hyperspectral imaging system designed for space applications. Dong et al. (2026) proposed a recognition model for unsound maize kernels based on HSI technology, where the multilayer perceptron neural network achieved a recognition accuracy of 96.5%. Zheng et al. (2026) explored a synergistic convolution and linear attention transformer architecture to enhance maize kernel damage classification performance and designed a modular hyperspectral image-level classification framework compatible with existing pixel-level feature extraction networks. Yang et al. (2024) proposed a maize kernel classification method that integrates HSI with a CNN embedded with spectral and spatial attention modules. Huang et al. (2024) constructed an enhanced ResNeSt-E network by integrating hyperspectral imaging technology with deep learning methods for detecting mechanical damage in maize kernels. Wang et al. (2022) proposed a maize kernel defect detection method based on the watershed algorithm and a dual-channel CNN model.

The above references demonstrated HSI captures band-continuous spectral reflection characteristics, which can effectively characterize variations in the internal physicochemical components of grain kernels. However, HSI lacks spatial structural details and remains difficult to fully characterize grain properties when used independently. Therefore, the effective fusion of spatial structural advantages of RGB images and intrinsic physicochemical spectral information of hyperspectral data has become a critical research direction for further improving the recognition accuracy of unsound maize kernels. Against this background, this paper constructs a multimodal maize kernel dataset containing RGB images and hyperspectral data, and proposes a novel MHENet. Supported by CMPE feature alignment, BMA modeling, and EAG fusion mechanism, the proposed model achieves efficient collaborative representation and complementary utilization of multimodal information, thereby substantially enhancing the recognition performance for complex and easily confused defective maize kernel samples.

## 3 Methodology

**Figure 1:**
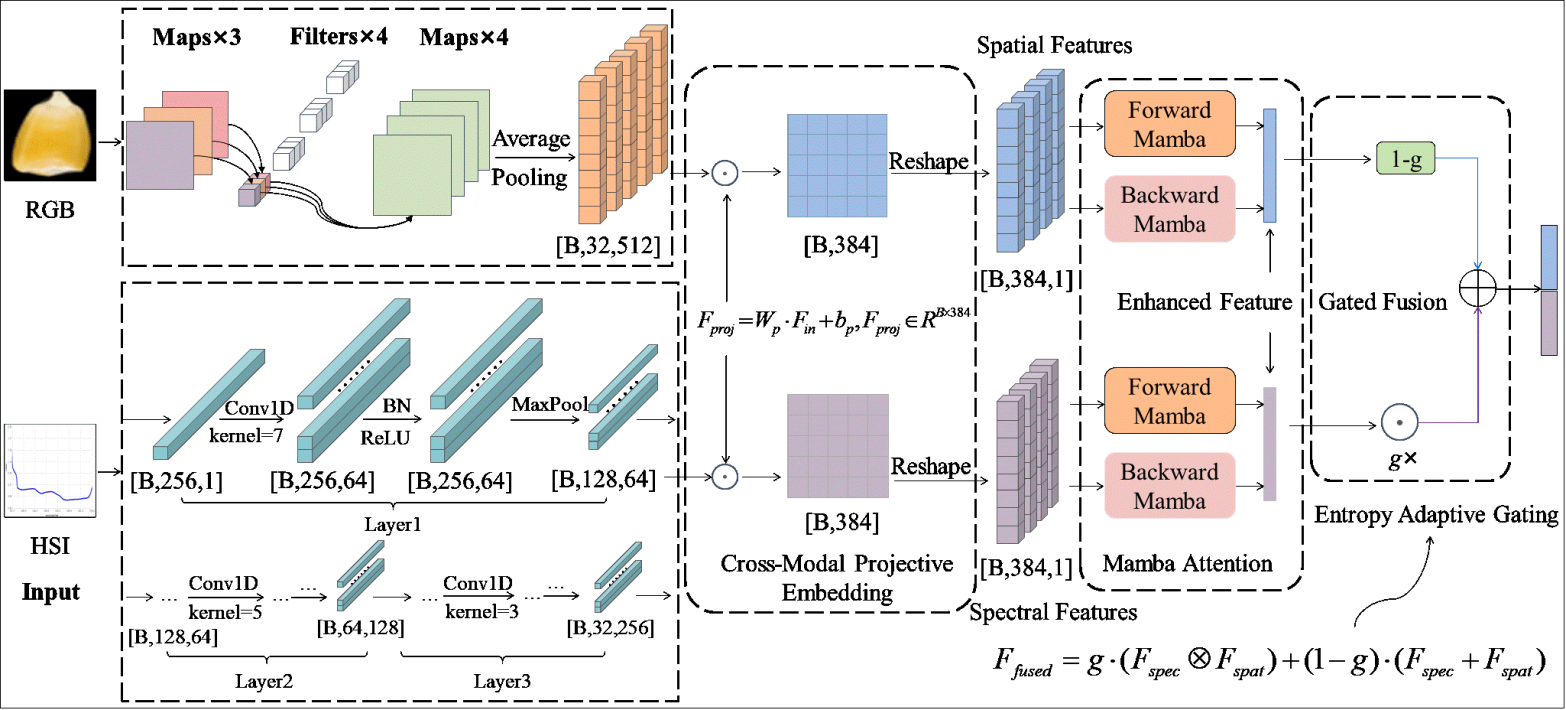
Overall architecture of the proposed MHENet for unsound maize kernel classification.

To fully exploit the complementary information between HSI and RGB image data, this paper proposes the MHENet for classifying unsound maize kernels. By jointly learning spectral features and spatial structural features, the designed network achieves deep fusion of multimodal information, thereby effectively improving the fine-grained discriminative ability for unsound maize kernel recognition. The overall network architecture of MHENet is illustrated in **Figure 1**.

As depicted in **Figure 1**, the proposed MHENet mainly consists of five key modules: a hyperspectral feature extraction module, a spatial feature extraction module, a CMPE, a BMA, and an EAG. At the model input stage, HSI is first fed into the spectral branch, where a 1DCNN-3 is adopted to extract representative spectral features. Meanwhile, the corresponding RGB sample images are transmitted to the spatial branch for the extraction of spatial structural and morphological information via a lightweight convolutional network. Subsequently, the two types of extracted modal features are projected into a unified latent feature space through the CMPE module, realizing accurate alignment of heterogeneous cross-modal features. To further model the long-range dependencies among different feature dimensions, the aligned multimodal features are separately imported into the BMA module for comprehensive global context feature enhancement. Finally, the EAG module is utilized to achieve dynamic adaptive weighted fusion of the enhanced multimodal information, and the optimized fused features are subsequently input into the classifier to complete the final category prediction of unsound maize kernels.

### 3.1 Architecture of the 1DCNN-3 model

**Figure 2:**
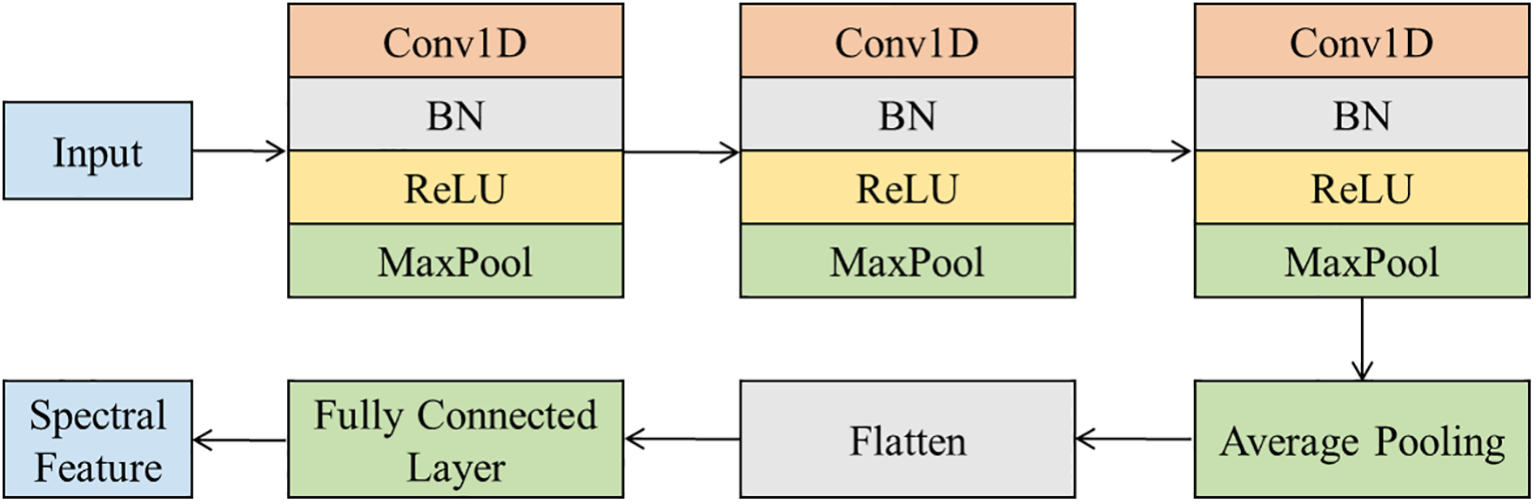
Structure of the 1DCNN-3 hyperspectral feature extraction network.

HSI data consists of numerous contiguous spectral bands, where each band corresponds to the material reflectance characteristics at a specific wavelength, presenting strong inherent continuity and correlation along the spectral dimension. To fully mine and effectively utilize the inherent structural spectral features of HSI data, this paper designs a spectral feature extraction module based on 1DCNN-3, and the structural diagram is shown in **Figure 2**.

As shown in **Figure 2**, the hyperspectral sequence is first fed into 1D convolutional layers for local spectral feature extraction. In the first stage, a relatively large convolution kernel (kernel size = 7) is adopted to convolve the spectral sequence, which aims to capture spectral variation patterns over a wide spectral range. Benefiting from the large receptive field, the model can effectively learn global correlation relationships among different spectral regions. Subsequently, batch normalization (BN) and ReLU activation function are employed to enhance the stability and generalization of feature distribution, followed by max-pooling downsampling to compress feature dimensionality and reduce redundant computational consumption. In the second stage, the convolution kernel size is reduced to 5 to further extract fine-grained spectral features, while the number of convolution channels is increased to strengthen the nonlinear feature representation capability of the network. By constructing a layer-by-layer deepened network structure, the model can gradually learn hierarchical feature representations from low-level original spectral information to high-level abstract semantic features. In the third stage, the convolution kernel size is further reduced to 3 to capture finer local spectral variation details, thereby effectively enhancing the model’s ability to distinguish subtle spectral differences among different defective maize kernel samples.

To eliminate the influence of sequence length on fully connected layers and retain global statistical information at the feature level, the adaptive global average pooling (AGAP) is introduced at the end of the network to compress the spatial dimension to 1, generating a feature vector of ${\mathbb{R}}^{B\times 512}$. A linear fully connected layer is then adopted to project the pooled features into a unified low-dimensional feature space, and the specific calculation formula is defined as follows (Li et al., 2024):

(1) $$F_{specout}=W_{spec}\cdot GAP(X_{stage3})+b_{spec},F_{spe\text{co}ut}\in {\mathbb{R}}^{B\times 512}$$

where $F_{specout}$ denotes the output spectral feature vector after projection; $W_{spec}$ represents the trainable weight matrix of the fully connected layer for spectral feature projection; *GAP* (·) denotes the global average pooling operation; $X_{stage3}$ represents the output feature map of the third convolutional stage in 1DCNN-3; $b_{spec}$ denotes the trainable bias vector corresponding to $W_{spec}$; ${\mathbb{R}}^{B\times 512}$ represents the feature dimension.

The 1DCNN-3 is specifically selected as the hyperspectral feature extractor due to its perfect theoretical matching with hyperspectral sequence features and inherent advantages over other lightweight sequential architectures. Hyperspectral data is a continuous one-dimensional physical sequence with hierarchical feature distribution (global spectral trends and local band differences). The three-layer progressive convolution design (kernel sizes = 7, 5, 3) of 1DCNN-3 can theoretically realize multi-scale feature extraction, which simultaneously captures global spectral correlation and local subtle spectral variations that determine the internal physicochemical differences of defective maize kernels. Compared with LSTM, 1DCNN adopts full convolution with linear computational complexity, avoiding the quadratic complexity and long-range dependency vanishing defects of recurrent networks in modeling long hyperspectral sequences. In contrast to ResNet1D (redundant residual connections for 1D spectral data) and unbalanced 1DCNN variants, 1DCNN-2 lacks sufficient nonlinear fitting ability due to insufficient receptive fields, while 1DCNN-4/5 has theoretical risks of overfitting and gradient disappearance caused by excessive depth. Therefore, 1DCNN-3 achieves the optimal theoretical balance between feature representation capability and model lightweight.

### 3.2 Architecture of the MobileNetV4-Small model

**Figure 3:**
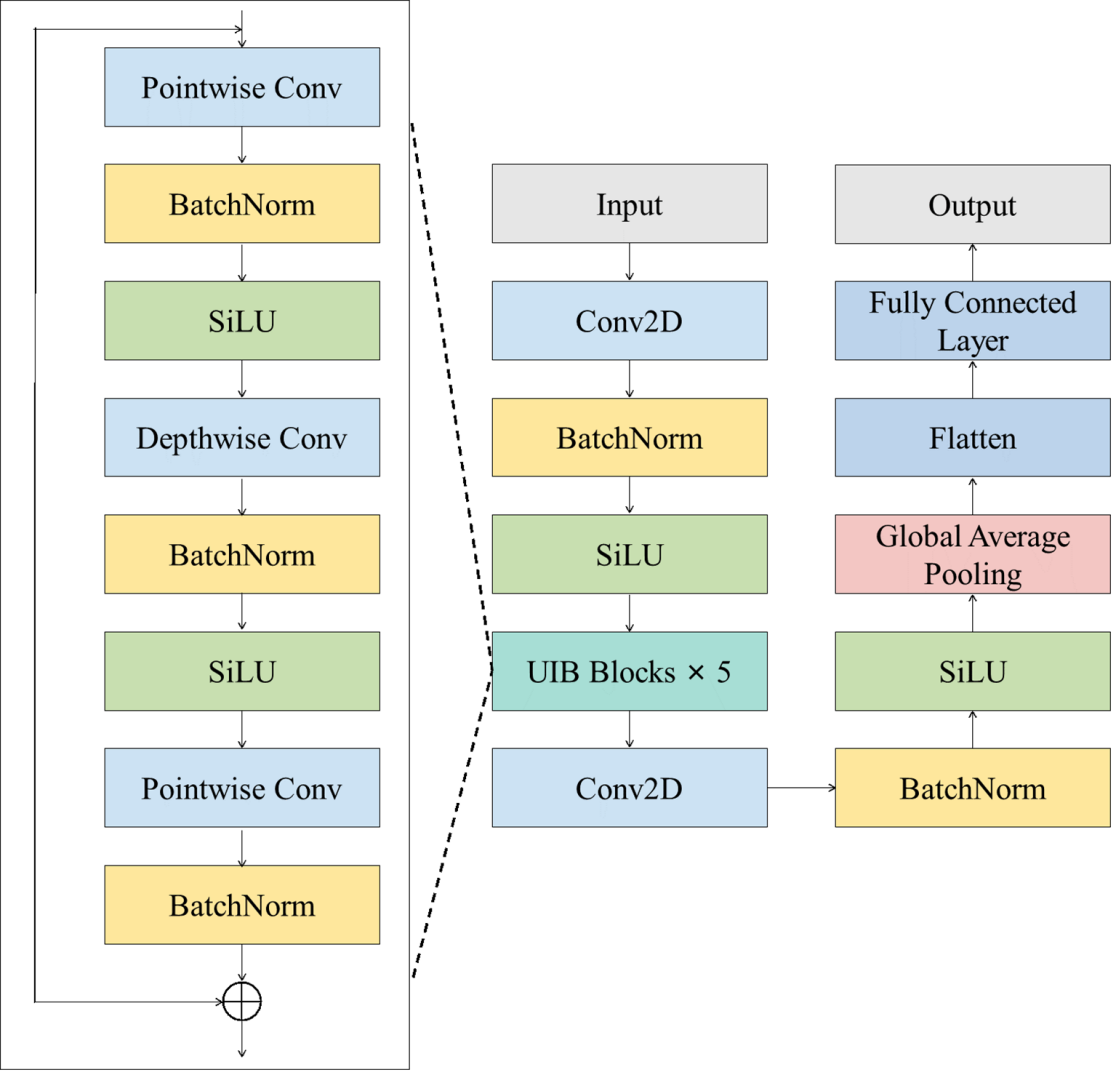
Structure of the MobileNetV4-Small spatial feature extraction network.

In addition to intrinsic spectral physicochemical information, the spatial structure, surface texture distribution, and morphological characteristics of maize kernel samples also contain critical discriminative information for defect classification. Therefore, this paper adopts the lightweight convolutional neural network MobileNetV4-Small as the spatial feature extraction branch, and structural diagram is illustrated in **Figure 3**.

MobileNetV4-Small decomposes standard convolution into depthwise convolution and pointwise convolution via depthwise separable convolution operations, which substantially reduces the overall computational complexity and parameter count of the network. The input RGB images are first processed by multi-layer convolutional modules to perform hierarchical feature extraction, and multi-scale convolutional structures are employed to learn spatial semantic information ranging from local textures to global structures. As the network depth increases, the extracted feature maps are gradually evolved from low-level original texture information to high-level abstract spatial semantic representations. At the end of the network, global average pooling (GAP) is employed to compress the extracted spatial features into fixed-dimensional global feature vectors, which are further mapped into 512-dimensional spatial feature representations through a linear transformation layer. The specific calculation process is expressed as (Jin et al., 2024):

(2) $$F_{spatout}=W_{spat}\cdot MNv4(X_{spat})+b_{spat},F_{spatout}\in {\mathbb{R}}^{B\times 512}$$

where $F_{spatout}$ denotes the output spatial feature vector after linear transformation; $W_{spat}$ represents the trainable weight matrix of the linear projection layer for spatial features; MNv4(·) denotes the feature extraction process of the MobileNetV4-Small network; $X_{spat}$ represents the input RGB image sample for spatial feature extraction; $b_{spat}$ denotes the trainable bias vector corresponding to $W_{spat}$; ${\mathbb{R}}^{B\times 512}$ represents the feature dimension.

The structural design not only realizes effective feature dimensionality reduction but also preserves key spatial structural and texture information of maize kernels, thus improving the overall computational efficiency of the model. The lightweight network architecture can significantly reduce computational overhead and deployment cost while maintaining high feature extraction and recognition performance. Furthermore, the network possesses excellent nonlinear feature expression capability, which can provide rich and reliable spatial semantic support for subsequent multimodal feature fusion and fine-grained defect classification.

The MobileNetV4-Small is selected as the spatial feature extractor based on solid theoretical advantages over other lightweight CNNs for maize kernel appearance feature extraction. Theoretically, MobileNetV4-Small adopts depthwise separable convolution, which decomposes standard convolution into spatial filtering and channel fusion, reducing computational complexity by nearly 1/*k* (*k* is channel number) compared with standard convolution, fundamentally meeting the lightweight requirements of on-site grain detection equipment. Different from MobileNetV3 (hard sigmoid activation causes theoretical feature loss) and ResNet18 (standard convolution redundancy), MobileNetV4-Small integrates inverted residual bottleneck and UIB blocks, which theoretically enhance fine-grained feature extraction (tiny mold spots, insect holes, micro-cracks) by expanding channel capacity and reducing information loss. Compared with EfficientNet-V2 and ConvNeXt series (compound scaling/large-kernel convolution theories are unsuitable for small-target grain defects), MobileNetV4-Small has a more appropriate receptive field and moderate theoretical complexity, avoiding parameter redundancy and feature irrelevance. Thus, MobileNetV4-Small is the optimal theoretical choice for RGB spatial feature extraction of maize kernels.

### 3.3 Design of the CMPE module

Spectral and spatial features are derived from two completely different data modalities, leading to substantial discrepancies in their inherent feature distribution characteristics and representation spaces. Direct multimodal feature fusion without prior alignment will inevitably cause severe modal mismatch, which seriously degrades subsequent fusion effectiveness and overall model recognition performance. To address this issue, this paper proposes a cross-modal projective embedding (CMPE) module. Within this module, the 512-dimensional features from both the spectral branch and the spatial branch are first mapped to a unified 384-dimensional embedding space via a linear projection layer. Through this approach, features from different modalities can be projected into the same semantic space, thereby reducing the distribution discrepancies between modalities.

(3) $$F_{proj}=W_{p}\cdot F_{in}+b_{p},\;\;\;\;F_{proj}\in R^{B\times 384}$$

where $F_{proj}$ denotes the projected feature vector in the unified latent space; *W_p_* represents the trainable weight matrix for cross-modal projection; *F_in_* denotes the input unimodal feature before projection; *b_p_* represents the trainable bias vector corresponding to *W_p_*.

To further stabilize the training process, a learnable scale parameter ${\text{λ}}_{i}$ is introduced into the module to dynamically adjust the projected features, so as to avoid gradient explosion or excessive feature amplitude in the early training stage. The feature distribution is fine-tuned via diagonal matrix multiplication as follows:

(4) $$F_{aligned}=diag(\lambda_{1},\lambda_{2},\ldots ,\lambda_{384})\cdot F_{proj}$$

where $F_{aligned}$ denotes the final aligned cross-modal feature; $\text{diag}(\lambda_{1},\lambda_{2},\ldots ,\lambda_{384})$ represents the diagonal matrix composed of learnable scale parameters ${\text{λ}}_{i}(i=1,2,\ldots ,384)$; ${\text{λ}}_{i}$ denotes the learnable scaling factor for feature amplitude adjustment.

After processing by the CMPE module, the hyperspectral and image features are not only unified to $R^{\left\{\text{B}\times 384\right\}}$ in dimension, but their distribution characteristics and semantic expressions also achieve preliminary regularized alignment within the common manifold space, denoted as $F_{spec}^{\prime }$ and $F_{spat}^{\prime }$.

The core role of the CMPE module is to achieve cross-modal feature alignment, enabling information from different modalities to be represented in a unified feature space. Compared with fusion methods based on direct feature concatenation, the proposed method effectively reduces semantic bias between modalities and improves the efficiency of subsequent feature interaction. In addition, the low-dimensional embedding space representation further reduces the overall computational complexity of the model and optimizes practical inference efficiency.

### 3.4 Design of the BMA module

**Figure 4:**
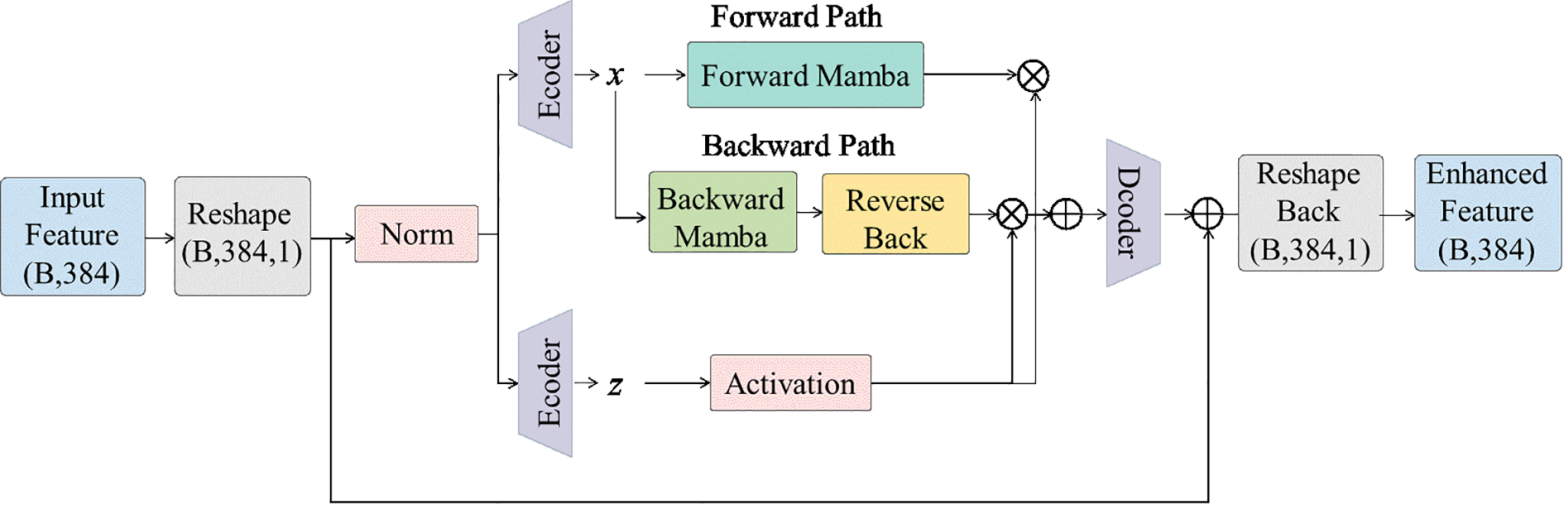
Structure of the BMA module.

After completing cross-modal feature alignment via the CMPE module, it is still essential to effectively model the long-range global dependencies among different feature dimensions to enhance feature discriminability. Traditional convolutional structures primarily focus on local information, while the standard self-attention mechanism, although capable of capturing global relationships, exhibits high computational complexity. To address the issue, this paper introduces a BMA module for feature enhancement, as illustrated in **Figure 4**.

The BMA module is constructed based on the state space model (SSM), which captures complex contextual correlations among feature dimensions through efficient sequence modeling. Specifically, the aligned feature vector is first regarded as a one-dimensional sequential signal and fed into the forward Mamba module to conduct sequential modeling, so as to capture forward directional dependencies between feature dimensions. Subsequently, the feature sequence is reversely arranged and input into the backward Mamba module to learn reverse contextual dependency information. Finally, the forward and backward enhanced feature representations are fused to obtain the final feature with comprehensive bidirectional global contextual information. This bidirectional sequential modeling strategy enables the model to fully utilize dual-directional contextual information and comprehensively capture global correlation relationships among multimodal features. Furthermore, compared with the conventional Transformer self-attention structure, the Mamba-based architecture possesses superior computational efficiency and shows unique advantages in processing long-range sequence features. With the introduction of the BMA module, the proposed network can effectively strengthen the capability of nonlinear feature representation while maintaining low computational consumption.

### 3.5 Design of the EAG module

**Figure 5:**
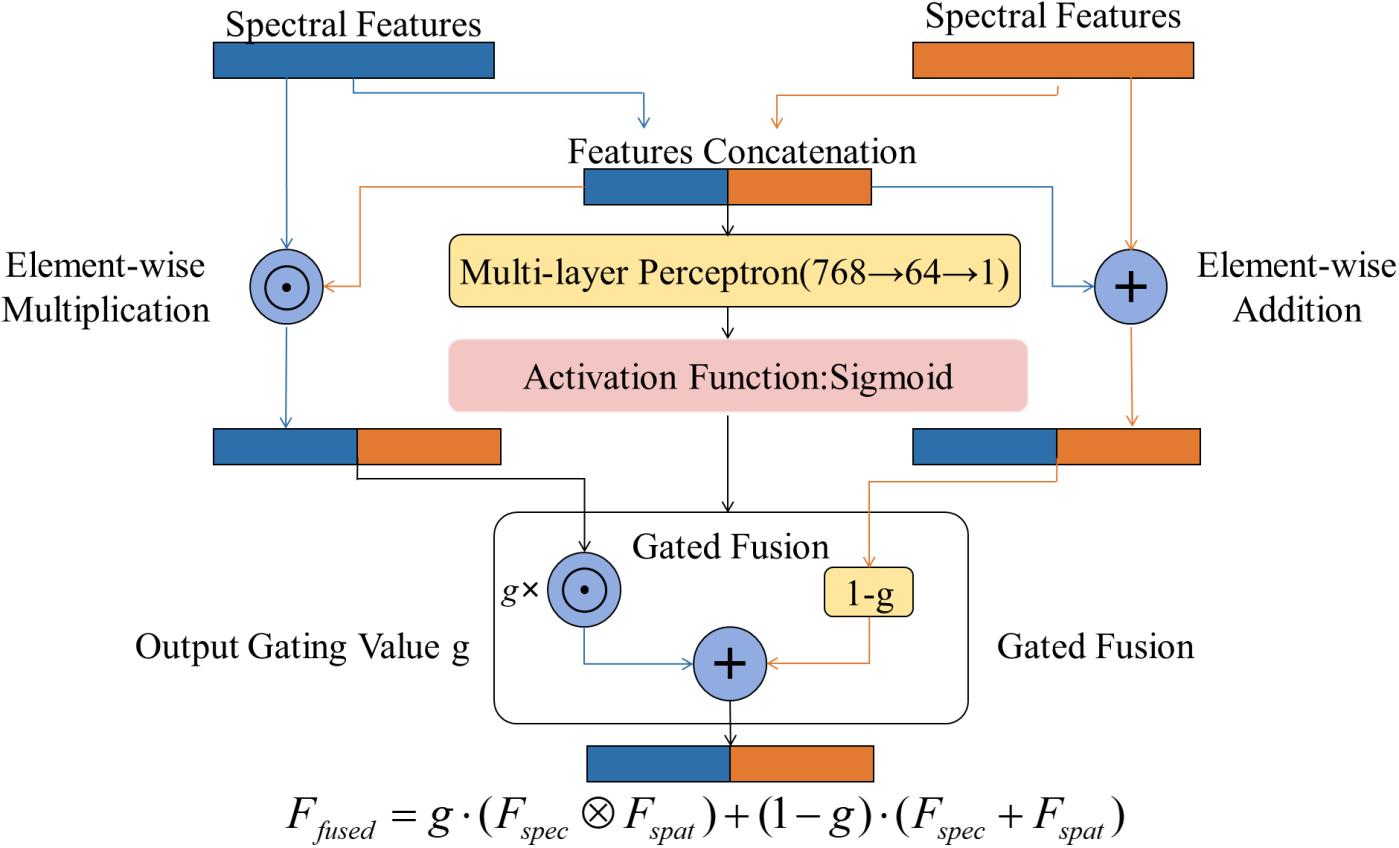
Structure of the EAG fusion module.

To achieve effective fusion of multimodal information, this paper proposes an EAG fusion module, as illustrated in **Figure 5**.

As shown in **Figure 5**, the proposed EAG module dynamically adjusts the contribution weights of different modalities during the fusion process according to the semantic information of input features. First, the two enhanced features vectors, $F_{spec},F_{spat}\in {\mathbb{R}}^{B\times 384}$, are concatenated along the channel dimension to form a 768-dimensional joint feature representation. The joint feature is then fed into a bottleneck multi-layer perceptron (MLP) with a ReLU activation function, which significantly compresses the feature dimensionality while preserving key feature information. Such a design further reduces the overall computational complexity of the model and drives the network to extract core entropy information that quantitatively characterizes the uncertainty and reliability of each individual modality.

(5) $$H_{joint}=ReLU(W_{1}\cdot \text{Concat}([F_{spec},F_{spat}])+b_{1})$$

where $H_{joint}$ denotes the hidden feature of the joint multimodal feature after compression; ReLU(·) represents the rectified linear unit activation function; $W_{1}$ denotes the trainable weight matrix of the first fully connected layer in the bottleneck MLP; Concat([·]) represents the feature concatenation operation along the channel dimension; $F_{spec}$ denotes the enhanced spectral feature after BMA module processing; $F_{spat}$ represents the enhanced spatial feature after BMA module processing; $b_{1}$ denotes the trainable bias vector corresponding to $W_{1}$.

Subsequently, a linear layer combined with the Sigmoid activation function is adopted to generate a scalar adaptive gating weight g, and is defined as:

(6) $$\begin{array}{c} g=Sigmoid(W_{2}\cdot H_{joint}+b_{2}) \end{array}$$

where Sigmoid(·) denotes the Sigmoid activation function for gating weight generation; $W_{2}$ represents the trainable weight matrix of the second fully connected layer in the bottleneck MLP; $b_{2}$ represents the trainable bias vector corresponding to $W_{2}$.

The final multimodal feature fusion strategy integrates both nonlinear multiplicative cross-interaction and linear additive residual connection between the two modalities, and the unified fusion formula is expressed as:

(7) $$\begin{array}{c} F_{fused}=g\cdot (F_{spec}⊙F_{spat})+(1-g)\cdot (F_{spec}+F_{spat}) \end{array}$$

where ⊙ denotes the Hadamard product. When the gating weight g approaches 1, the model relies on nonlinear cross-correlation information between spectral and spatial image features for final classification decision-making. When *g* approaches 0, the model switches to a linear residual fusion mode, which fully preserves the independent and original feature information extracted from both spectral and spatial branches. Benefiting from this adaptive fusion mechanism, the model can dynamically switch and optimize the fusion strategy according to the feature distribution characteristics of different maize kernel samples, thereby sufficiently exploiting the inherent complementarity between multimodal information and improving overall fusion quality.

## 4 Experiments

### 4.1 Dataset fabrication

The multimodal maize kernel image acquisition system consists of dual acquisition units: visible-light RGB imaging and hyperspectral data. Notably, the RGB camera and hyperspectral imaging (HSI) camera adopt asynchronous acquisition mode, rather than real-time synchronous triggering. To guarantee strict one-to-one correspondence between RGB images and HSI data for each individual maize kernel, we adopt a fixed-position sequential acquisition strategy combined with physical location calibration and unique sample coding. Specifically, each single maize kernel is fixed at a precisely marked position on the acquisition stage without any displacement. First, the RGB image of the fixed kernel is captured, and the sample is assigned a unique ID. Then, the hyperspectral data of the same kernel at the identical position is collected sequentially, and the same unique ID is bound to the HSI data. Through this fixed-position, sequential, coded pairing method, we completely eliminate the risk of sample mismatch caused by asynchronous acquisition, ensuring the pairing accuracy and reliability of all bimodal samples in the dataset.

**Figure 6:**
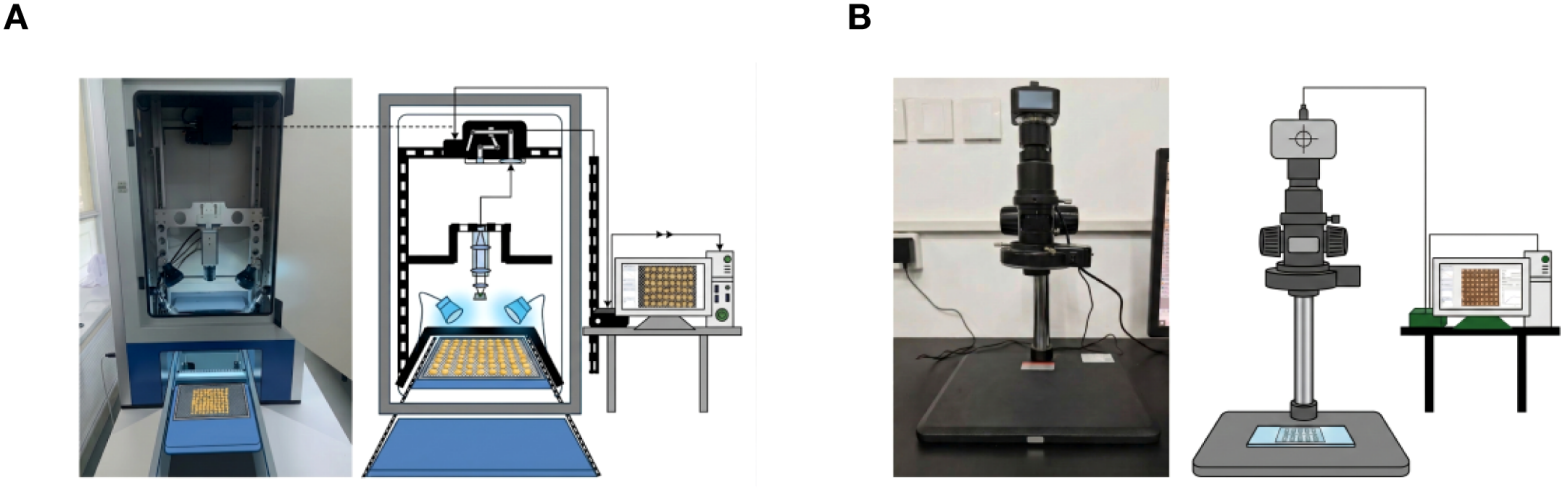
Schematic of the acquisition equipment. **(a)** Hyperspectral acquisition equipment. **(b)** RGB image acquisition equipment.

For RGB imaging, a BETICAL SZ7D-T510 industrial camera (BETICAL Optoelectronics Technology Co., Ltd., Suzhou, China) is adopted, with a resolution of 2592 × 1944 pixels, to capture the appearance features of maize kernels under uniform annular LED illumination (Shenzhen Helios Optoelectronics Co., Ltd., Shenzhen, China). For hyperspectral data, a GaiaSorter push-broom system (Zolix Instruments Co., Ltd., Beijing, China) is utilized, covering a spectral range of 900 nm – 1700 nm. Prior to image acquisition, black-and-white calibration is conducted to eliminate noise and mitigate uneven illumination, thereby ensuring data stability. The hyperspectral and RGB image acquisition equipment are presented in **Figure 6a** and **Figure 6b**, respectively.

**Figure 7:**
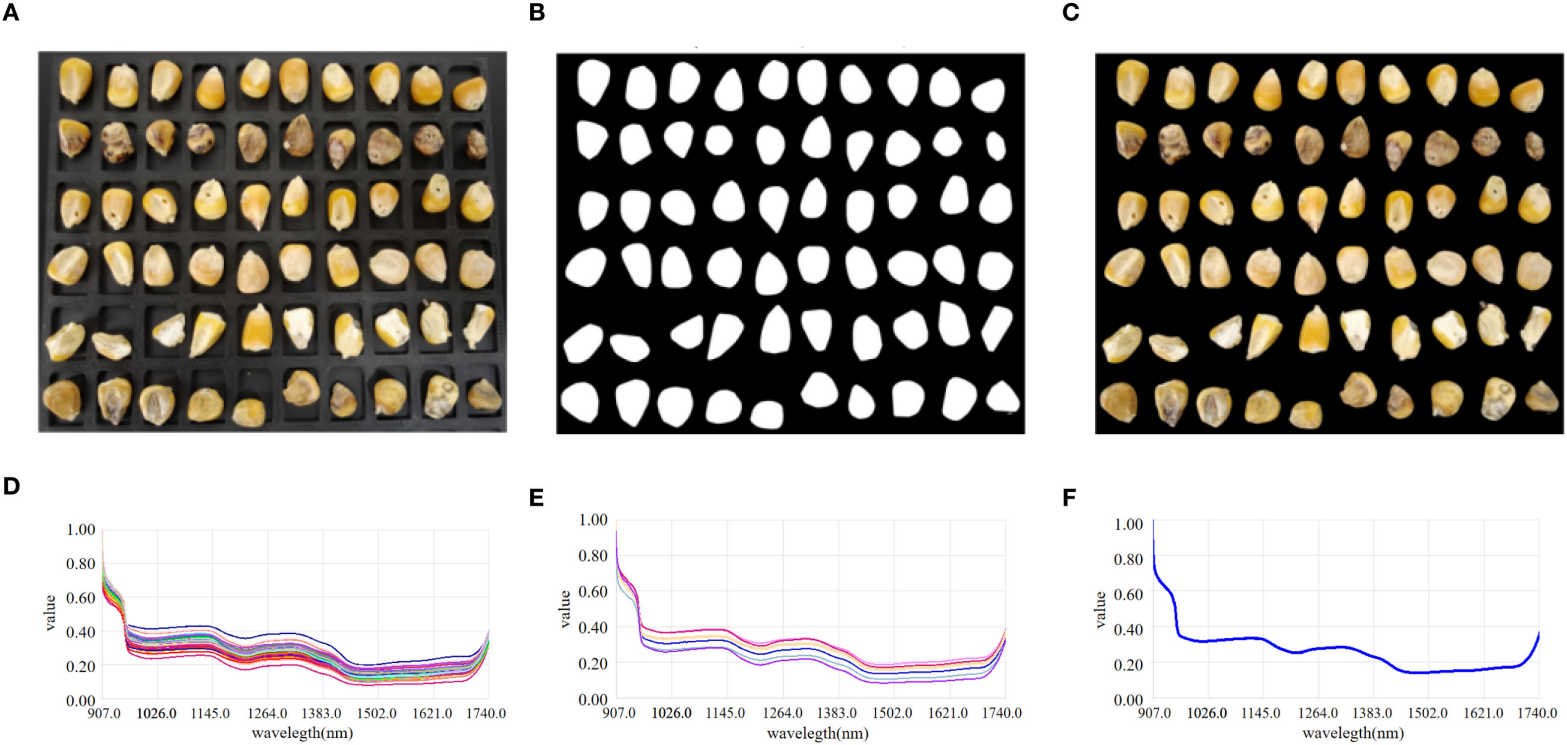
Process of maize kernel image segmentation and spectral feature extraction. **(a)** Raw maize kernel image. **(b)** Kernel geometric reconstruction mask. **(c)** Background-free single maize kernel image. **(d)** Raw spectral map. **(e)** Valid pixel spectral curves. **(f)** Average spectral feature curve.

The maize image preprocessing process mainly consists of three stages: color space conversion, threshold segmentation, and geometric reconstruction. The detailed processes of image preprocessing and spectral feature extraction are exhibited in **Figures 7a–f**. **Figure 7a** shows the raw maize kernel image, **Figure 7b** presents the kernel geometric reconstruction mask, **Figure 7c** displays the background-free single maize kernel image, **Figure 7d** shows the raw hyperspectral map, **Figure 7e** illustrates the valid pixel spectral curves, and **Figure 7f** exhibits the final average spectral feature curve of a single kernel. First, the acquired RGB images are converted into the hue-saturation-value (HSV) color space, and the value channel is extracted for subsequent processing. Subsequently, the Otsu adaptive thresholding algorithm is adopted to binarize the V-channel, and a morphological closing operation with a 9 × 9 kernel is integrated to connect internal fractures of the maize kernels. To obtain a complete morphological mask of maize kernels, the algorithm first extracts the contours of independent connected regions in the binary image, then applies the convex hull algorithm to calculate the minimum convex polygon for each connected region. Finally, a bitwise and operation is performed between the generated solid convex hull mask and the original RGB image, thereby completing the preprocessing of single maize kernel images.

The processing of hyperspectral data (covering a spectral range of 900 nm – 1700 nm with a raw total of 256 bands) and the extraction of single-kernel spectral features are mainly divided into spatial dimension extraction and spectral dimension aggregation. To mitigate the overfitting risk caused by high raw spectral dimensionality, the successive projections algorithm (SPA) is employed for HSI band selection and dimensionality reduction. SPA automatically screens 64 optimal informative bands from the original 256 bands by eliminating redundant, noisy, and highly collinear spectral channels, which drastically reduces spectral dimensionality while fully retaining the key physicochemical characteristic information related to maize kernel defects. First, based on the morphological mask generated earlier, the boundaries of maize kernels in the hyperspectral data cube are located, followed by segmentation and cropping to obtain independent single-kernel hyperspectral image samples. Subsequently, within the region of interest (ROI) of a single kernel, the raw spectral reflectance curves of all valid pixels covered by the mask are extracted. To eliminate noise induced by surface scattering, uneven illumination, and mixed edge pixels resulting from the physical shape of the kernels, interquartile range outlier removal and Savitzky-Golay filtering processes are performed on the initial pixel-level spectral dataset within a single ROI. Finally, the arithmetic mean of the filtered valid pixel spectra is calculated along the spatial dimension to generate a smooth average spectral curve. This curve serves as a one-dimensional spectral feature vector that represents the overall physical and chemical properties of the individual maize kernel, which is directly used for the construction and analysis of subsequent classification models.

In the sample labeling stage, images were manually screened and labeled based on appearance integrity and defect types. Samples were divided into sound kernels and unsound kernels, where the former refers to normal kernels and the latter includes five types of defective kernels: insect-damaged kernels, broken kernels, diseased kernels, heat-damaged kernels, and moldy kernels. Each category contains 1000 sample pairs (consisting of RGB images and HS data), resulting in a total of 6000 sample pairs. A double-cross-check method was employed during the labeling process to review boundary samples, thereby reducing the impact of human-induced mislabeling. For samples with inconspicuous defect areas but abnormal overall morphology, a comprehensive judgment was made by integrating information such as kernel edges, surface texture, and color variations to enhance the consistency and reliability of the labels.

### 4.2 Parameter settings

All experiments are conducted under the PyTorch framework. The workstation used in this paper is configured with an Intel Xeon Gold 5220R CPU and an NVIDIA A4000 GPU. The detailed training hyperparameters are listed in **Table 1**. To ensure the fairness of comparative experiments, all networks involved in this paper adopt a unified training strategy and are trained from random initialization without the use of any pre-trained weights.

**Table 1:** Hyperparameter settings for model training.

Hyperparameter	Configuration
Optimizer	Adam
Learning Rate (LR)	1e^-3^
Batch Size	64
Epoch	120
Weight Decay	1e^-4^
Loss Function	Cross-entropy cosine annealing from initial LR to 1e^-6^

To fully verify the consistency and reproducibility of experimental results, all classification comparison experiments, core module ablation experiments and multimodal fusion validation tests were independently repeated 5 times under exactly the same hardware environment and hyperparameter settings. All reported quantitative metrics adopt the mean value and standard deviation of five repeated runs, which eliminates the interference caused by network random initialization and random data partition.

### 4.3 Performance and analysis

To comprehensively verify the effectiveness of the proposed MHENet in the task of unsound maize kernel recognition, this paper conducted quantitative and qualitative comparative analyses from three aspects: unimodal feature extraction, multimodal fusion strategies, and key module construction, thereby systematically evaluating the recognition accuracy, computational efficiency, and structural rationality of the model.

**Figure 8:**
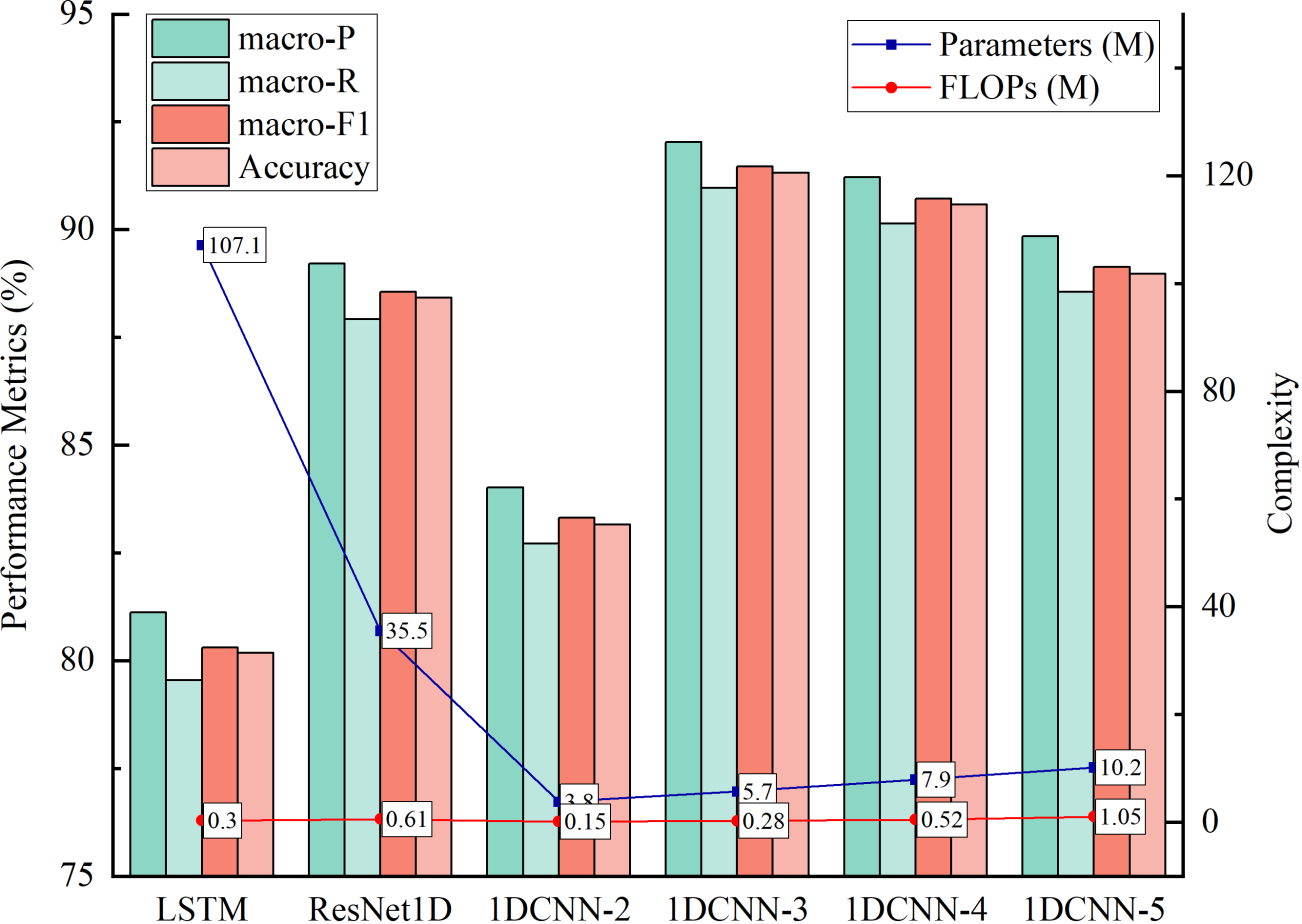
Performance comparison of different spectral feature extractors on hyperspectral data.

Hyperspectral data characterizes the differences in internal chemical components of maize kernels by means of reflectance across continuous spectral bands. To select the optimal extractor applicable to hyperspectral features, this paper compared six types of sequence models under the same dataset and training configuration, including LSTM, ResNet1D, and 1DCNNs with varying depths. The performance comparison of different spectral feature extraction models is presented in **Figure 8**. Due to its sequential structure, LSTM fails to efficiently capture long-range dependencies between spectral bands and exhibits high computational redundancy, achieving an accuracy of only 80.18% and a computational cost as high as 107.1 million (M). ResNet1D effectively mitigates gradient vanishing through residual connections, raising its accuracy to 88.42% and reducing its computational cost to 35.5 MFLOPs. However, its parameter counts of 0.61 MFLOPs still poses challenges for lightweight deployment. In contrast, 1DCNNs exhibit stronger adaptability in hyperspectral sequence modeling.

Among the 1DCNN series models, 1DCNN-3 exhibits the best overall performance, achieving an accuracy of 91.32%, which is 2.90% higher than that of ResNet1D. Meanwhile, its computational cost is only 5.7 MFLOPs and its parameter count is 0.28 M, thus achieving a dual breakthrough in both accuracy and computational efficiency. When the network depth is further increased to 4 or 5 layers, the model shows a significant performance degradation: the accuracies of 1DCNN-4 and 1DCNN-5 drop to 90.58% and 88.97%, respectively, while the computational cost and parameter count continue to rise. The above results indicate that there is no linear relationship between network depth and recognition performance in hyperspectral sequence modeling. Excessively deep networks tend to cause overfitting and feature redundancy, thereby reducing the discriminative ability for fine-grained defects. Therefore, 1DCNN-3 is selected as the backbone feature extraction network for the hyperspectral modality.

**Figure 9:**
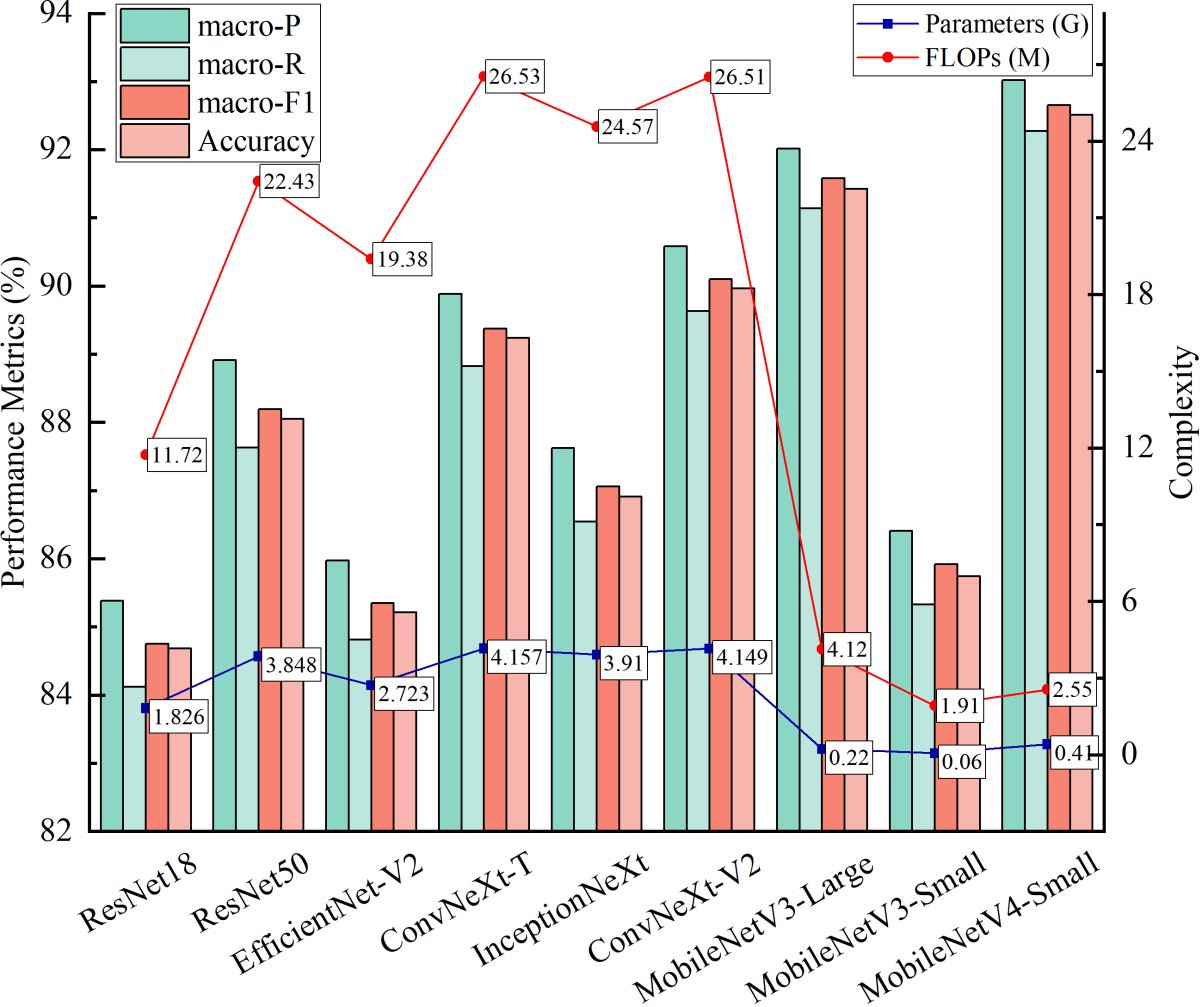
Comparison of average performance for image classification.

The proposed model extracts appearance defect features (e.g., surface texture, morphology, and color) of maize kernels from RGB images. To select the optimal backbone network for spatial feature extraction, this paper compared nine mainstream models, including traditional residual networks, new-generation convolutional architectures, and lightweight dedicated networks. The experimental results are shown in **Figure 9**. Traditional large-scale convolutional networks exhibit obvious bottlenecks in this task: ResNet50 has 22.43 M parameters and a computational cost of 3.848 GFLOPs, achieving an accuracy of only 88.05%. EfficientNet-v2 achieves only 85.21% accuracy with 19.38 M parameters. Models such as ConvNeXt and InceptionNeXt both have over 24 M parameters and a computational cost of nearly or above 4 GFLOPs, with a maximum accuracy of only 89.96%. Experiments indicate that general large-scale models fail to exert advantages in global modeling for fine-grained defect recognition of maize kernels, and are plagued by severe parameter redundancy and computational overhead.

MobileNetV3-Small achieved an accuracy of 85.74% with 1.91 M parameters and 0.06 GFLOPs, thus verifying the effectiveness of depthwise separable convolution. Among these models, MobileNetV4-Small exhibited the best overall performance, achieving an accuracy of 92.51% with only 2.55 M parameters and 0.41 GFLOPs. Compared with ResNet50, it reduced parameters by 88.63%, computational cost by 89.35%, and improved accuracy by 4.46%. Benefiting from its inverted bottleneck structure and efficient convolutional operators, the model could accurately capture subtle defects such as mold and insect damage. It can be concluded that the matching degree between the network structure and task characteristics is far more important than simply expanding the model scale. Therefore, this paper adopted MobileNetV4-Small as the backbone network for the RGB image modality.

**Figure 10:**
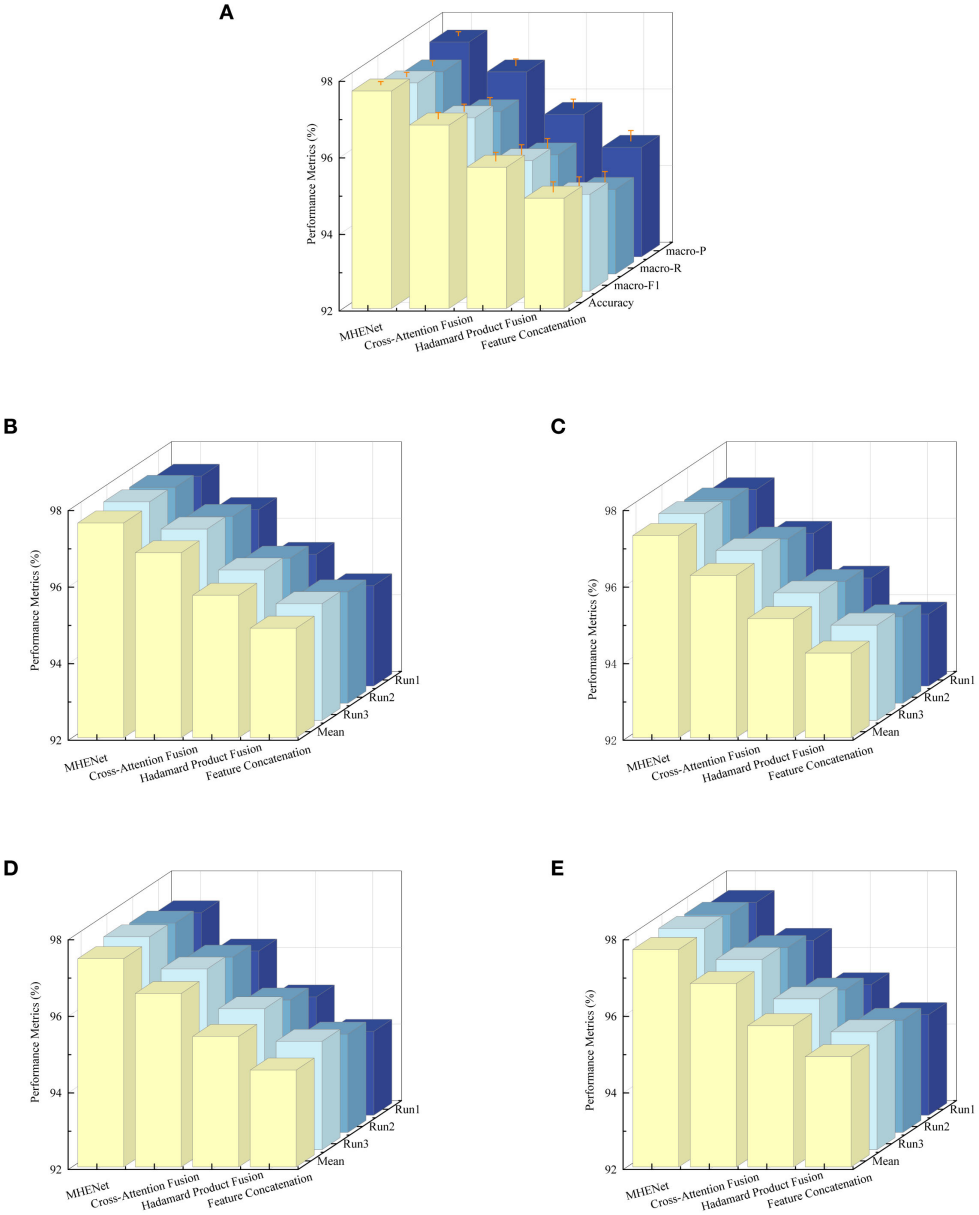
Performance comparison of multimodal fusion methods. **(a)** Average performance metrics of different fusion strategies. **(b)** Macro-P of four independent experiments. **(c)** Macro-R of four independent experiments. **(d)** Macro-F1 of four independent experiments. **(e)** Accuracy of four independent experiments.

Based on the optimal unimodal backbone networks selected above, this paper further conducted comparative experiments on multimodal fusion strategies, including simple feature concatenation, Hadamard product, cross-attention, and the proposed MHENet architecture. The overall performance and repeatability results of different fusion strategies are shown in **Figures 10a–e**. **Figure 10a** compares the average performance metrics (macro-P, macro-R, macro-F1, and accuracy) of four fusion methods, while **Figures 10b–e** present the macro-P, macro-R, macro-F1, and accuracy values of four independent repeated experiments, respectively. Basic feature concatenation fusion achieved an accuracy of 94.88% and a macro-F1 score of 94.53% with 3.72 M parameters and a computational cost of 0.74 GFLOPs, which was 2.37% higher than that of the optimal unimodal model. This verified that RGB and hyperspectral information exhibited strong complementarity and could effectively improve discriminative accuracy. Hadamard product fusion realized shallow feature interaction through element-wise multiplication, increasing the accuracy to 95.69%. However, limited by its local interaction mechanism, its performance improvement was constrained. Transformer-based cross-attention fusion boosted the accuracy to 96.79% by modeling long-range dependencies, but introduced significant computational redundancy, with parameters and computational cost increasing to 5.34 M and 1.12 GFLOPs, respectively, making it challenging to meet the lightweight deployment requirements in agricultural scenarios.

The proposed MHENet model achieved deep multimodal feature fusion through the three-level collaborative mechanism consisting of CMPE, BMA, and EAG, achieving a final accuracy of 97.68% and a macro-F1 score of 97.44%. Compared with the cross-attention fusion method, the proposed MHENet increased the accuracy by 0.89%, while reducing the parameter count to 4.87 M and the computational cost to only 0.96 GFLOPs. Experimental results demonstrate that the proposed method can efficiently fuse multimodal information while avoiding the complexity bottleneck associated with conventional attention mechanisms, thus achieving collaborative optimization between classification accuracy and computational efficiency.

**Figure 11:**
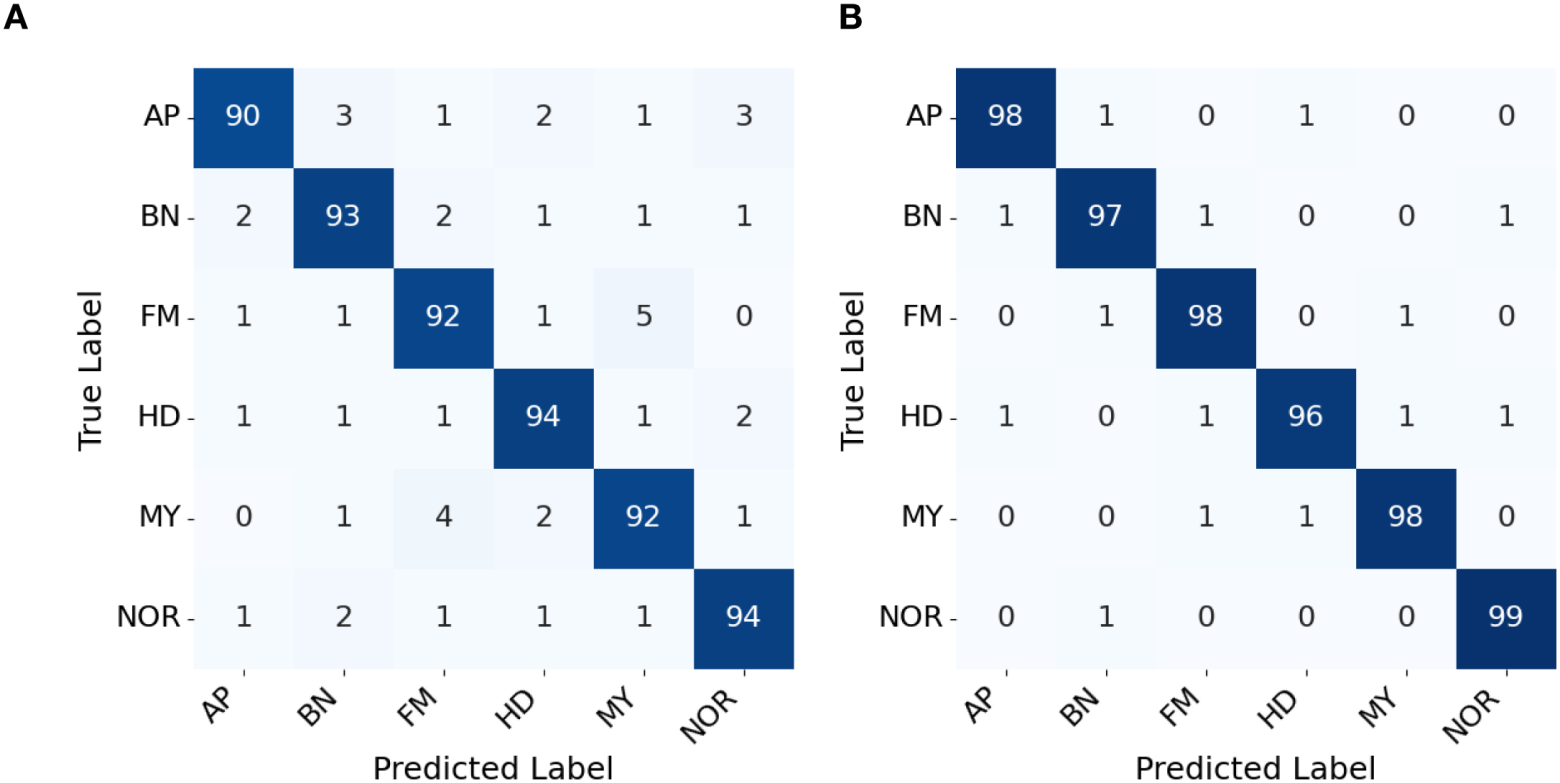
Confusion matrices. **(a)** MobileNetV4-Small. **(b)** MHENet.

To intuitively reveal the influence of multimodal fusion on classification decisions, the confusion matrices of the MobileNetV4-Small (unimodal RGB model) and MHENet (multimodal fusion model) were compared in the experiments. The horizontal and vertical axes of the confusion matrices represent five maize kernel categories: NOR (normal kernels), AP (aflatoxin-polluted kernels), BN (broken kernels), HD (hollow-decayed kernels), and MY (moldy kernels). The confusion matrix of the unimodal MobileNetV4-Small is shown in **Figure 11a**, and the confusion matrix of the proposed MHENet is presented in **Figure 11b**. When only RGB images were used, the model exhibited obvious confusion between moldy kernels and diseased kernels, mainly due to the high similarity in color distribution and surface texture between these two defect categories. After introducing hyperspectral information and adopting the MHENet fusion strategy, the recognition accuracy of both categories increased to above 97%, and the number of cross-category misclassified samples decreased significantly.

**Figure 12:**
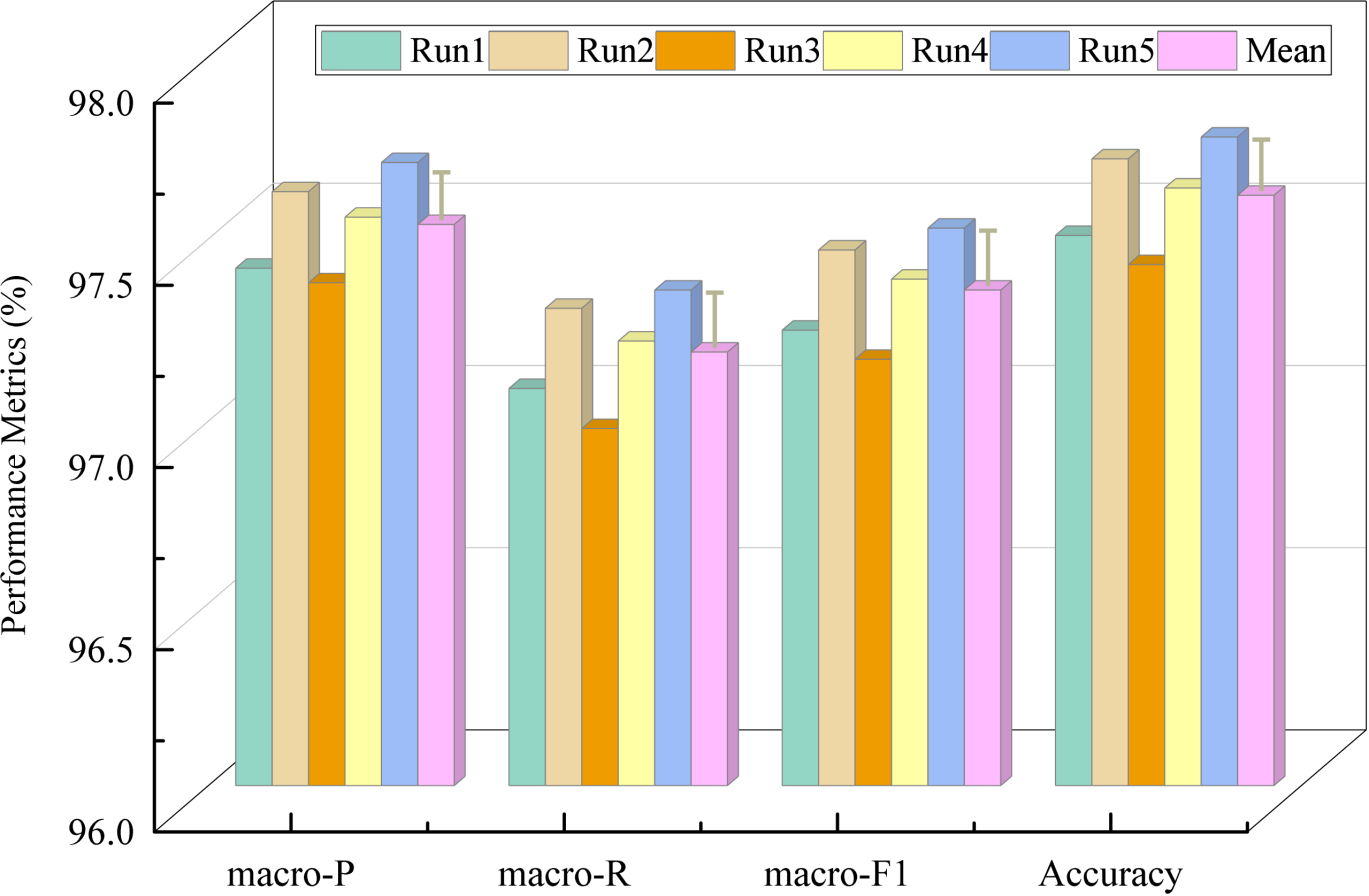
Results of five-fold cross-validation.

To verify the stability and generalization ability of MHENet and avoid performance fluctuations caused by random dataset splitting, five-fold cross-validation was adopted in the experiments. The experimental results are shown in **Figure 12**. Under five independent training-test divisions, the accuracy of MHENet was stably distributed in the range of 97.43% to 97.78%, with an average accuracy of 97.62% and a standard deviation of only 0.14. The extremely low performance fluctuation exhibited by the model in five-fold cross-validation indicates excellent stability and reliable generalization ability under the current dataset splitting and experimental settings.

To statistically validate the authenticity and reliability of the performance superiority of the proposed MHENet, a paired two-sample *t*-test was performed on the classification results obtained from five-fold cross-validation. The statistical analysis demonstrates that the recognition accuracy of MHENet is significantly higher than that of all unimodal baselines, traditional fusion strategies, and existing multimodal models. In particular, compared with the cross-attention fusion method (the second-best performing method), the accuracy improvement of MHENet reaches 0.89% with a significant statistical difference. Meanwhile, the performance gaps between MHENet and single-modal models (MobileNetV4-Small, 1DCNN-3) are extremely significant. These statistical results confirm that the performance advantages of the proposed model originate from the rationality of the framework design rather than random data partitioning or experimental fluctuations, which rigorously verifies the superiority and stability of MHENet.

To further compare the applicability and performance differences of various attention mechanisms in the constructed dual-modal unsound maize kernel classification task, comparative ablation experiments were conducted between the proposed BMA module and four mainstream classic attention mechanisms, namely SE, ECA, CA, and CBAM. The corresponding quantitative experimental results are summarized in **Table 2**. As typical channel-only attention mechanisms, SE and ECA cannot effectively balance spatial feature representation and long-range dependency modeling, resulting in relatively low classification accuracies of 95.82% and 96.14%, respectively. Although CA and CBAM introduce additional spatial information modeling branches to compensate for the deficiencies of single-channel attention, their feature modeling capability is inherently limited by the local receptive field of convolutional operations. Both methods still fail to efficiently capture long-range spectral dependencies among hyperspectral bands, only achieving classification accuracies of 96.59% and 96.95%, respectively. In comparison, the proposed BMA module realizes global bidirectional long-range feature enhancement with linear computational complexity. maintained a low computational cost of only 0.96 GFLOPs, the BMA module achieves the highest recognition accuracy of 97.68%. The proposed module significantly outperforms all other comparative attention modules, verifying that BMA is more suitable for the dual-modal collaborative feature enhancement task based on RGB and HSI data.

**Table 2:** Comparative analysis of attention mechanisms.

Attention mechanism	macro-P (%)	macro-R (%)	macro-F1 (%)	Accuracy (%)	FLOPs (G)	Parameters (M)
SE	95.84	95.31	95.57	95.82	0.88	4.22
ECA	96.12	95.67	95.89	96.14	0.86	4.15
CA	96.58	96.11	96.34	96.59	0.93	4.48
CBAM	96.93	96.48	96.70	96.95	0.98	4.76
BMA	97.61	97.28	97.44	97.68	0.96	4.87

**Figure 13:**
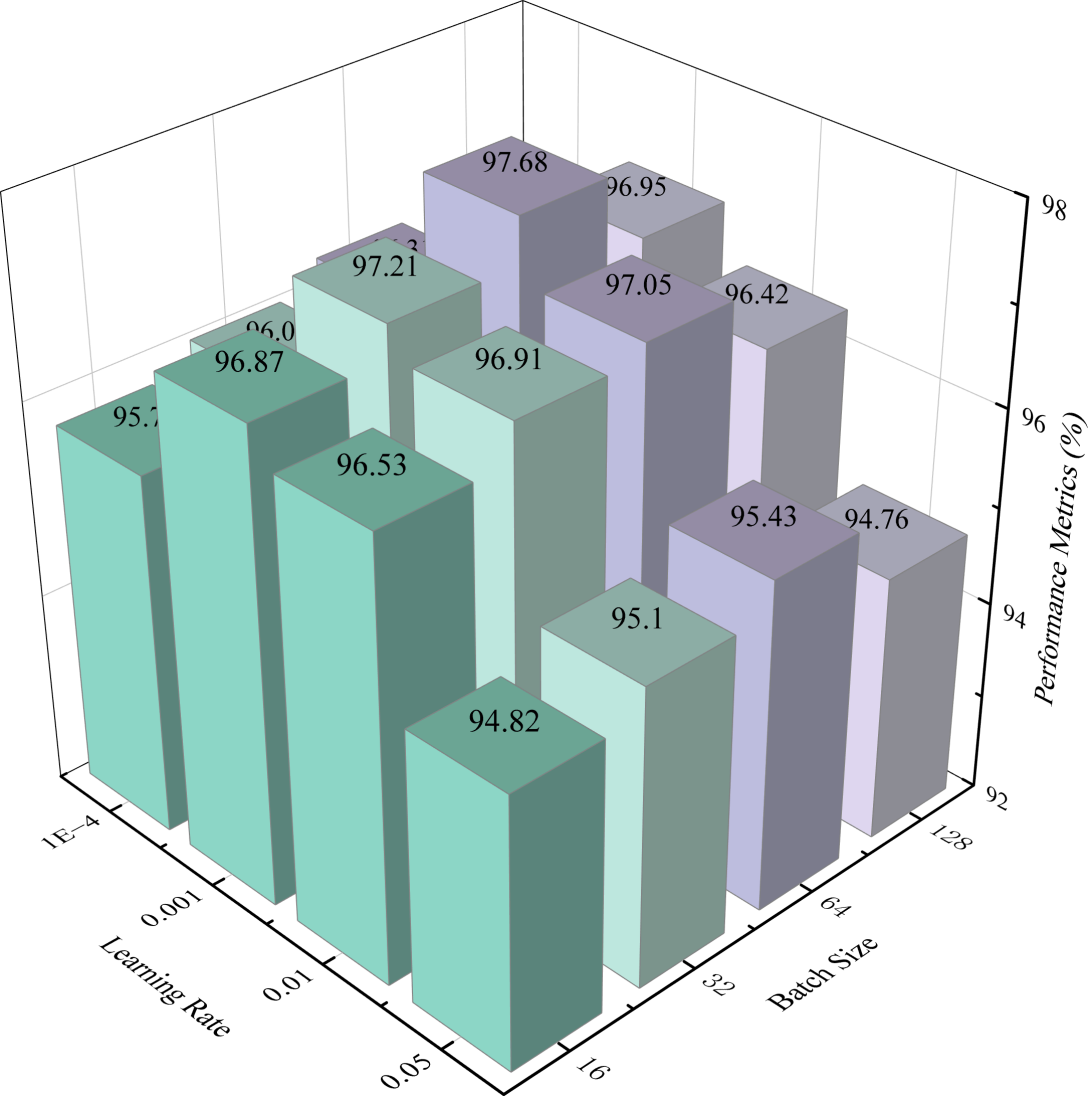
Parameter sensitivity analysis.

To determine the optimal training parameters for the model, this paper conducted full factorial comparative experiments on two key hyperparameters: batch size and learning rate. The batch size was set to 16, 32, 64, and 128, while the learning rate was set to 0.05, 0.01, 0.001, and 0.0001. The experimental results are shown in **Figure 13**. These two hyperparameters exhibit obvious coupling effects, and their combined impact directly determines the model’s training performance and convergence efficiency. An excessively large learning rate (e.g., 0.05) caused severe training oscillations and impeded model convergence, with the highest classification accuracy reaching only 95.23%. When the learning rate was appropriately reduced to 0.01, the training stability of the model was significantly improved, and the classification accuracy increased to 96.87%. However, a further reduction in the learning rate (e.g., 0.001 or 0.0001) led to extremely slow convergence, prolonged the training cycle, and even resulted in failure of model convergence.

In terms of batch size selection, a small batch size (e.g., 16) introduced excessive random fluctuations during the model training process, which led to unstable training dynamics and a relatively low classification accuracy of only 94.51%. As the batch size increased to 64, the model achieved optimal training stability and classification performance, with the classification accuracy reaching its peak value of 97.68%. When the batch size further exceeded 64 (e.g., 128), the generalization capability of the model degraded noticeably, and the corresponding classification accuracy declined to 96.15%. This phenomenon occurred because an excessively large batch size reduced the diversity of training samples participating in each iteration, limiting the model’s ability to learn fine-grained feature differences among various defective maize kernel categories and ultimately deteriorating the overall classification performance.

Based on the comprehensive comparative analysis of the foregoing hyperparameter experimental results, the optimal training configuration of the proposed model was finally determined as a batch size of 64 and a learning rate of 0.01. This optimal configuration not only guarantees satisfactory training stability and high classification accuracy for the model but also achieves a favorable balance of training efficiency. In-depth analysis shows that the superior performance of MHENet stems from the rational combination of bimodal complementation and adaptive feature learning: RGB images provide accurate localization for surface defects such as breakage and insect damage, while hyperspectral data captures implicit physicochemical changes caused by mold, disease, and heat damage. The synergistic feature learning mechanism eliminates the limitation of single-modal information, which fundamentally reduces the misclassification of fine-grained defective samples with similar appearances. For real-world grain quality inspection systems, this configuration enables the model to meet the efficiency requirements of online detection, laying a solid and reliable foundation for the practical deployment and industrial application of the model in actual agricultural grain quality inspection scenarios.

Furthermore, to quantitatively evaluate the practical deployment capability of the proposed model, the computational training time and inference latency are tested under the experimental hardware environment. For the training process, MHENet requires a total training duration of 3.2 hours over 120 epochs, with an average of 96 seconds consumed per epoch. In terms of inference efficiency, the single-sample inference latency is only 2.8 milliseconds (ms), and the batch inference latency reaches 18.5 ms at the optimal batch size of 64. The low training overhead and high inference speed fully satisfy the real-time detection requirements of actual grain quality inspection and online sorting systems. The superior computational efficiency and ultra-low latency further verify the feasibility of MHENet for edge-side deployment and industrial application in on-site grain depots and processing production lines.

To further validate the competitiveness and superiority of the proposed MHENet, we comprehensively compare it with several recent state-of-the-art multimodal agricultural vision models tailored for RGB-hyperspectral fusion and grain quality detection, including DualCMNet and SSMSFuse. DualCMNet adopts 1DCNN and MobileNetV3 for dual-modal feature extraction, but its static fusion mechanism fails to capture long-range spectral dependencies, resulting in a lower accuracy of 96.35%. SSMSFuse focuses on spectral-spatial image fusion rather than classification, making it incompatible with lightweight deployment requirements. In contrast, the proposed MHENet integrates cross-modal alignment, bidirectional long-range feature enhancement, and entropy-driven adaptive fusion, achieving the highest classification accuracy of 97.68% with only 4.87 M parameters and 0.96 GFLOPs. The comparison results demonstrate that MHENet achieves a better balance between recognition precision and computational efficiency than existing state-of-the-art multimodal agricultural vision models, further verifying its novelty and practical competitiveness for unsound maize kernel classification.

### 4.4 Ablation experiments

To clarify the individual contribution of each core module and their internal collaborative mechanism in the proposed model, ablation experiments were conducted in this paper, with conventional feature concatenation fusion adopted as the baseline for comparative evaluation. As presented in **Table 3**, the baseline model achieved a classification accuracy of 94.88%, along with a computational cost of 0.74 GFLOPs and a parameter count of 3.72 M. The independent introduction of the CMPE module improved the model classification accuracy to 95.96%. This module effectively alleviates cross-modal discrepancies by unifying the heterogeneous feature space of different data modalities, and the additional computational overhead brought by module embedding is negligible, verifying its excellent practical application potential. When the BMA module was deployed independently, the model accuracy increased to 96.48%, yielding the most significant performance improvement among all single-module experiments. This performance advantage is attributed to the powerful capability of the BMA module in modeling long-range spectral dependencies and spatial structure correlations, which substantially enhances the model’s discriminative ability to capture subtle feature differences between various defect categories. The standalone utilization of the EAG module improved the classification accuracy to 95.58%. The EAG module can dynamically adjust the contribution weight of each modality according to the real-time discriminative performance of input features, effectively suppressing invalid noise interference from a single modality while only causing a slight increase in overall computational complexity.

**Table 3:** Ablation experiments of core modules.

CMPE	BMA	EAG	macro-P (%)	macro-R (%)	macro-F1 (%)	Accuracy (%)	FLOPs (G)	Parameters (M)
✗	✗	✗	94.86	94.21	94.53	94.88	0.74	3.72
✓	✗	✗	96.02	95.41	95.71	95.96	0.83	4.15
✗	✓	✗	96.54	96.01	96.27	96.48	0.91	4.62
✗	✗	✓	95.63	95.02	95.32	95.58	0.79	3.95
✓	✓	✗	97.12	96.76	96.94	97.21	0.92	4.71
✗	✓	✓	96.88	96.41	96.64	96.91	0.94	4.83
✓	✗	✓	96.41	95.93	96.17	96.45	0.87	4.38
✓	✓	✓	97.61	97.28	97.44	97.68	0.96	4.87

The experimental results indicate that there is an obvious positive synergistic effect among the modules. Combining CMPE and BMA yields an accuracy of 97.21%, since aligned features further enhance the effect of attention modeling. When CMPE, BMA, and EAG are all enabled, MHENet achieves the optimal accuracy of 97.68%, with computational cost and parameters controlled at 0.96 GFLOPs and 4.87 M, respectively. The three core modules undertake the key functions of feature alignment, feature enhancement, and adaptive fusion respectively, forming a hierarchical and progressive collaborative optimization structure, which enables the model to achieve optimal performance in both recognition accuracy and computational efficiency.

The experimental results verify a distinct positive synergistic interaction among the three proposed core modules. The combination of the CMPE and BMA modules achieves a classification accuracy of 97.21%, benefited from the fact that cross-modal feature alignment further improves the effectiveness of subsequent global attention modeling. When the CMPE, BMA, and EAG modules are fully integrated and activated, the proposed MHENet model reaches the optimal classification accuracy of 97.68%, while the computational cost and parameter scale are well controlled at 0.96 GFLOPs and 4.87 M, respectively. Each core module undertakes an indispensable function, including heterogeneous feature alignment, global feature enhancement, and multimodal adaptive fusion, thereby constructing a hierarchically progressive collaborative optimization framework. This elaborate design enables the model to achieve an excellent balance between high recognition accuracy and lightweight computational efficiency, which fully validates the rationality of the module design and the effectiveness of the internal synergistic mechanism.

Overall, the ablation experimental results sufficiently demonstrate that each individual core module contributes positively to boosting the classification performance of the model. Moreover, the multi-module collaborative combination further improves the model’s robustness and generalization capability. From a mechanistic perspective, CMPE eliminates the mismatch of heterogeneous feature distribution to lay a unified semantic foundation for feature fusion, BMA breaks the limitation of convolutional local receptive fields to capture long-range spectral dependencies critical for fine-grained distinction, and EAG adaptively suppresses single-modal noise to maximize bimodal complementarity. This hierarchical progressive design not only optimizes classification accuracy but also controls computational overhead strictly, which is highly compatible with the lightweight deployment requirements of actual grain inspection equipment. The optimized model can effectively reduce manual misjudgment during grain storage and circulation, providing reliable technical support for the practical intelligent detection and industrial application of unsound maize kernel quality inspection.

## 5 Conclusion

This paper proposed a multimodal hybrid enhancement network (MHENet) for unsound maize kernel classification, which was specifically designed for collaborative recognition based on dual-modal information (RGB visible light and HSI data). Experimental results demonstrated that the proposed MHENet can effectively fuse external phenotypic features and internal spectral physicochemical features, achieving a recognition accuracy of 97.68% for the six-category unsound maize kernel recognition task. Constrained by lightweight network architecture and high inference efficiency requirements, the proposed model achieved comprehensive performance optimization and can stably distinguish various fine-grained defective samples, including insect-damaged, broken, diseased, heat-damaged, and moldy maize kernels. The high classification accuracy, low computational cost, and strong stability of MHENet make it fully applicable to real-world grain quality inspection systems, such as rapid detection at grain procurement stations, online sorting of processing lines, and safety monitoring of grain storage, which can effectively reduce post-harvest grain losses and improve the efficiency of quality grading. In addition, the proposed MHENet model would be directly extended to quality inspection, defect identification, and grading tasks of other major grain crops.

Future work will focus on expanding dataset diversity (maize varieties, planting origins, moisture contents, and long-term storage gradients), adapting to complex environmental variability (strong/weak illumination, dust interference, and uneven kernel placement), and improving model robustness.

## Data Availability

The original contributions presented in the study are included in the article/supplementary material. Further inquiries can be directed to the corresponding author.
